# The lung microbiome in patients with pneumocystosis

**DOI:** 10.1186/s12890-017-0512-5

**Published:** 2017-12-04

**Authors:** J. Kehrmann, B. Veckollari, D. Schmidt, O. Schildgen, V. Schildgen, N. Wagner, M. Zeschnigk, L. Klein-Hitpass, O. Witzke, J. Buer, J. Steinmann

**Affiliations:** 1 Institute of Medical Microbiology, University Hospital Essen, University Duisburg-Essen, Hufelandstr. 55, 45122 Essen, Germany; 2 Institute of Pathology, Kliniken der Stadt Köln gGmbH, Hospital of the University of Witten/Herdecke, Cologne, Germany; 3 Institute of Human Genetics, Faculty of Medicine, University of Duisburg-Essen, Essen, Germany; 4 Biochip Laboratory, Institute for Cell Biology–Tumor Research, University of Duisburg-Essen, Essen, Germany; 5 Department of Infectious Diseases, University of Duisburg-Essen, Essen, Germany; 6 Present Address: Institute of Clinical Hygiene, Medical Microbiology and Infectiology, Klinikum Nürnberg, Paracelsus Medical University, Prof.-Ernst-Nathan-Str. 1, 90419 Nürnberg, Germany

**Keywords:** *Pneumocystis jirovecii*, Pneumocystosis, Pulmonary microbiome

## Abstract

**Backround:**

*Pneumocystis jirovecii* pneumonia (PCP) is an opportunistic fungal infection that is associated with a high morbidity and mortality in immunocompromised individuals. In this study, we analysed the microbiome of the lower respiratory tract from critically ill intensive care unit patients with and without pneumocystosis.

**Methods:**

Broncho-alveolar fluids from 65 intubated and mechanically ventilated intensive care unit patients (34 PCP+ and 31 PCP- patients) were collected. Sequence analysis of bacterial 16S rRNA gene V3/V4 regions was performed to study the composition of the respiratory microbiome using the Illumina MiSeq platform.

**Results:**

Differences in the microbial composition detected between PCP+ and PCP- patients were not statistically significant on class, order, family and genus level. In addition, alpha and beta diversity metrics did not reveal significant differences between PCP+ and PCP- patients. The composition of the lung microbiota was highly variable between PCP+ patients and comparable in its variety with the microbiota composition of the heterogeneous collective of PCP- patients.

**Conclusions:**

The lower respiratory tract microbiome in patients with pneumocystosis does not appear to be determined by a specific microbial composition or to be dominated by a single bacterial species.

**Electronic supplementary material:**

The online version of this article (10.1186/s12890-017-0512-5) contains supplementary material, which is available to authorized users.

## Backround

*Pneumocystis jirovecii* is an opportunistic human pathogenic fungus causing pneumocystosis, a severe pulmonary infection occurring mainly in immunosuppressed patients. In the 1980s and 90s, pneumocystosis predominantly developed in HIV-patients with low CD4^+^ T cell counts and was classified as an acquired immunodeficiency syndrome (AIDS)-defining disease, associated with a high mortality rate [1]. Since the initiation of highly active antiretroviral therapy and the prophylactic administration anti-*Pneumocystis* drugs to patients at risk, the disease frequency has decreased in this patient group [2]. In recent years, pneumocystosis became a serious matter of concern in patients with other types of immunosuppression such as solid organ transplant recipients, patients with haematological malignancies or connective tissue diseases [3]. Studies based on serological data show that most children have contact with the fungus within the first years of life [4–6]. Pneumocystosis occurs in most cases as an unapparent infection in immunocompetent children and seems to permanently or intermittently colonize its host in low numbers [7].

The homeostasis of the composition of the normal respiratory tract flora is considered to be essential to prevent the expansion of pathogens. In case of the fungus *Aspergillus fumigatus*, dysbiosis due to underlying pulmonary diseases or immune system dysfunction has been reported to cause uncontrolled fungal colonization which may exacerbate into overt fungal disease [8]. Furthermore, it is widely accepted that microbiological communities are a major regulator of the immune system and that alterations in the lung and/or gut microbiota may allow exacerbations of existing chronic lung diseases and can trigger susceptibility to new infections [9]. Besides CD4^+^ T-cells, which play a major role in animal models of the host defence against *Pneumocystis* infection [10], other studies indicate that several other immune cells such as alveolar macrophages, dendritic cells, neutrophils and B lymphocytes are involved in the immunological response against this fungal pathogen [11]. In addition, the ecological determinants of the lung microbiome - immigration, elimination, and regional growth conditions all change dramatically during acute or chronic lung infection [12]. Very recently, it was shown that respiratory infection with *Pneumocystis murina* influences the alpha and beta diversity of the gut microbiota of CD4+ intact and CD4-depleted mice and resulted also in changes in taxa abundances indicating the role of a gut-lung axis during *Pneumocystis* infection [13].

To the best of our knowledge, studies of the human lung microbiome during *P. jirovecii* pneumonia are lacking so far. In this study, we evaluated the lung microbiota in broncho-alveolar lavages (BAL) from patients with pneumocystosis and critically ill patients without *Pneumocystis* pneumonia (PCP) by sequencing bacterial 16S rRNA amplicons in the V3/V4 regions. Our aim was to study if a specific lung microbiome exists in pneumocystosis patients in comparison to the lung microbiome of other critically ill intensive care unit (ICU) patients.

## Methods

### Study design

In this retrospective observational study, we analysed the microbiome of BAL samples from intensive care unit patients treated in the University Hospital Essen, Essen, Germany. Thirty -four BAL samples of pneumocystosis (PCP+) patients treated between 2013 and 2016 were included in the analysis. Diagnosis of pneumocystosis was done by reviewing medical records, evaluating radiological images and a positive DNA result for *P. jirovecii* in real-time PCR (Sacace, Como, Italy). Furthermore, 31 BAL samples from patients with negative *Pneumocystis jirovecii* PCR (PCP-) between 2015 and 2016 were used as control. Only the first episode of pneumocystosis and one sample per patient was included in the analysis.

The study was performed in accordance with the Declaration of Helsinki and no written informed consent was necessary due to the retrospective design of the study. It was approved by the Ethics Committee of the Medical Faculty of the University of Duisburg-Essen (no. 16–6948).

### DNA isolation and sequencing

DNA was isolated from broncho-alveolar lavage samples in clinical routine on daily practice using the Maxwell® 16 instrument (Promega, Madison, WI) with the Maxwell® 16 Tissue LEV Total RNA Purification Kit (Promega). DNA was stored at −20 °C until further processing. The V3/V4 region of the 16S rRNA gene was amplified with the 341F forward primer and the 785R primers from Klindworth et al. [14], with an Illumina adapter overhang nucleotide sequence added 5′ of the locus-specific sequences. The sequence of the primer overhangs used was from the Illumina 16S Metagenomic Sequencing Library Preparation Guide (www.illumina.com): forward primer overhang: 5′-TCGTCGGCAGCGTCAGATGTGTATAAGAGACAG and reverse primer overhang: 5′-GTCTCGTGGGCTCGGAGATGTGTATAAGAGACAG. PCR was performed using the following steps: 95 °C for 3 min and 30 cycles of 95 °C for 30 s, 60 °C for 30 s and 72 °C for 30 s with a final extension of 72 °C for 5 min. PCR samples were run on a 1% agarose gel with a 100 bp ladder to check for amplification efficacy. PCR products were cleaned up using the Qiagen PCR purification kit (Hilden, Germany) and eluted in 30 μl TE-Buffer. 2.5 μl of the purified PCR product was used as a template for the second round of PCR using the N5XX and N7XX index primer of the Nextera XT Index Kit (Illumina, San Diego, CA). Each sample had a unique combination of N5XX and N7XX indices. PCR was performed with the following setting: 95 °C for 3 min and 10 cycles of 95 °C for 30 s, 55 °C for 30 s and 72 °C for 30 s with a final extension of 72 °C for 5 min. PCR samples were run on a 1% agarose gel. For purification of the PCR products with the QIAGEN PCR Purification Kit, 6 individual pools were generated containing similar amounts of PCR products as estimated by agarose gel electrophoresis. PCR products were eluted in 20 μl TE buffer and the DNA concentration of the sample pools was measured using the Qubit High-Sensitivity Assay (LifeTechnologies, Carlsbad, CA). All 6 sample pools were then combined to yield a single pool, which was quantified by qPCR using the NEBNext Library Quant Assay (NEB, Ipswich, MA, USA), and loaded on the flow cell at a concentration of 12 pM. A PhiX control library was spiked in at 3 pM concentration to increase sequence diversity, as recommended by Illumina. Sequencing was performed using the Illumina MiSeq 600 cycle reagent kit v3 (Illumina, San Diego, CA), with 301 cycles for read 1 and 2 and 8 cycles for the two index reads.

### Preprocessing and data analysis

Demultiplexed paired-end fastq files generated by CASAVA (Illumina) and a mapping file were used as input files. Sequences were pre-processed, quality filtered and analysed using QIIME2 version 2017.8 and QIIME1 version 1.91 [15]. We used the DADA2 software package [16], wrapped in QIIME2, for modelling and correcting Illumina sequenced fastq files including removement of chimeras with the “consensus” method. Fastq files were processed by the qiime dada2 denoise-paired command. Due to decreasing quality scores of the sequences at the end, especially for the reverse reads, we truncated 20 bases of the forward and 80 bases of the reverse read, resulting in a remaining overlap of 35 bases in merged sequences. Sample collection of PCP+ patients including direct DNA-extraction was performed over a period of 4 years (2013–2016) and PCP- samples from the years 2013 and 2014 were lacking. We determined the sequence variants that were significantly different distributed between PCP+ of both periods (2013–2014 vs. 2015–2016) and excluded them from further analyses. Therefore, we filtered the merged sequences output of PCP+ patients, calculated the statistically significant sequence variants by Kruskal-Wallis one-way analysis of variance by using the group_significance.py QIIME script with *p* < 0.05 without correction for multiple testing. These sequence variants were filtered from all sequence variants in all samples by using the QIIME2 qiime feature-table filter features command. This step was done to reduce probability of errors caused by contaminating bacterial species during the process of DNA extraction in the time where PCP- patient DNA was not available.

For taxa comparisons, relative abundances based on all obtained reads were used. We used the QIIME2 q2-feature-classifier plugin and the Naïve Bayes classifier that was trained on the Greengenes13.8 99% OTUs full-length sequences. QIIME2 taxa barplot command was used for viewing the taxonomic composition of the samples.

Alpha and beta-diversity analyses were performed with the q2-diversity plugin in QIIME2 at a sampling depth of 1000. One PCP+ sample was excluded from these analyses due to a sequence frequency of 251. Alpha diversity was calculated by Shannon’s diversity index, observed OTUs, Pielou’s measure of species evenness and Faith’s Phylogenetic Diversity. Permutational multivariate analysis of variance (PERMANOVA) was used to analyse statistical differences in beta diversity with QIIME2. Principal coordinate analyses (PCoA) was performed based on unweighted and weighted UniFrac, Bray-Curtis and Jaccard distances in QIIME2 and visualized with the make_2d_plots.py script of QIIME 1.91. Kruskal-Wallis test was used for taxa comparisons, calculated in QIIME 1.91. Benjamini–Hochberg false discovery rate (FDR) correction was used to correct for multiple hypothesis testing.

## Results

BAL samples from 65 ICU patients (21 female, 44 male) were included in the study, each with one sample per patient. *P. jirovecii*-DNA was detected in 34 BAL samples from pneumocystosis patients and was undetectable in BALs from 31 PCP- ICU patients. All PCP+ patients exhibited lung infiltrates in chest radiography. In six BAL samples from the PCP+ group, cysts were found by immune fluorescence microscopy. The patient characteristics of all 65 patients are displayed in Table 1.

**Table 1:** Characteristics of PCP+ and PCP- patients

	PCP+	PCP-
Age in years, mean (min-max)	55 (18–86)	65 (31–86)
Sex		
Male	21	23
Female	13	8
Diagnosis		
Malignancy	12	12
- Hematooncological	8	6
- Solid tumor	3	6
Solid organ transplantation	3	2
HIV	7	0
Liver cirrhosis	6	1
Infection	4	6
Heart disease	2	6
Cerebral haemorrhage	0	2
Lung fibrosis	0	2

After processing of the demultiplexed fastq files with the DADA2 package we excluded amplicon sequence variants significantly different distributed between PCP+ patients from 2013 to 2014 and 2015–2016 from all samples. We obtained 577,610 sequences with a total of 2750 amplicon sequence variants from the 65 filtered samples. The mean sequence frequency was 8886 ± 9615 SD). One PCP+ sample was excluded from diversity analyses due to a read number < 1000/sample after processing.

**Fig. 1:**
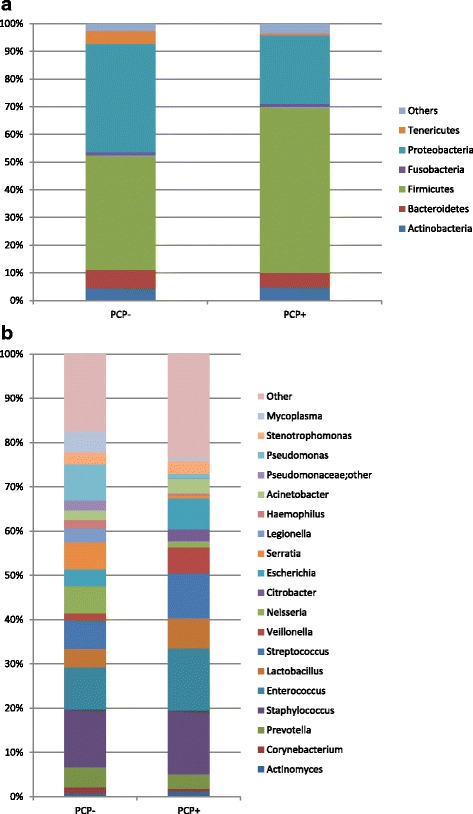
**a** + **b** Composition of the bacterial community at the phylum (**a**) and genus (**b**) level for PCP+ and PCP- samples. Phyla and genera with a minimum percentage of 1% are shown

The dominating phyla in PCP- and PCP+ patients were *Firmicutes* with 41.3% (± 34.2% SD) and 60.0% (± 31.8% SD) mean relative abundance and *Proteobacteria* with 39.1% (± 38.7% SD) and 24.8% (± 32.5% SD), respectively (Fig. 1a).The Phyla *Bacteroidetes*, *Actinobacteria*, *Tenericutes* and *Fusobacteria* contributed >1% and <10% to mean relative abundance of the total bacterial content. On the genus level, the most abundant genera were: *Staphylococcus* 13.1% (± 23.5% SD) for PCP- and 14.5% (± 23.2% SD) for PCP+ patients, *Enterococcus* 9.5% (± 24.5% SD) and 14.0% (± 29.1% SD), *Streptococcus* 6.5% (± 17.7% SD) and 10.2% (± 18.0% SD), *Escherichia* 3.3% (± 15.2% SD) and 7.0% (± 21.0% SD), *Serratia* 6.1% (± 23.3% SD) and 0.5 (± 2.9% SD), *Lactobacillus* 4.1% (± 8.7% SD) and 6.9% (± 15.8% SD) *Veillonella* 1.5% (± 4.0% SD) and 5.8% (± 12.7% SD), *Neisseria* 6.1% (± 16.0% SD) and 1.4% (± 5.3% SD), and *Prevotella* 4.6% (± 9.9% SD) and 3.2% (± 4.5% SD) (Fig. 1b).

**Fig. 2:**
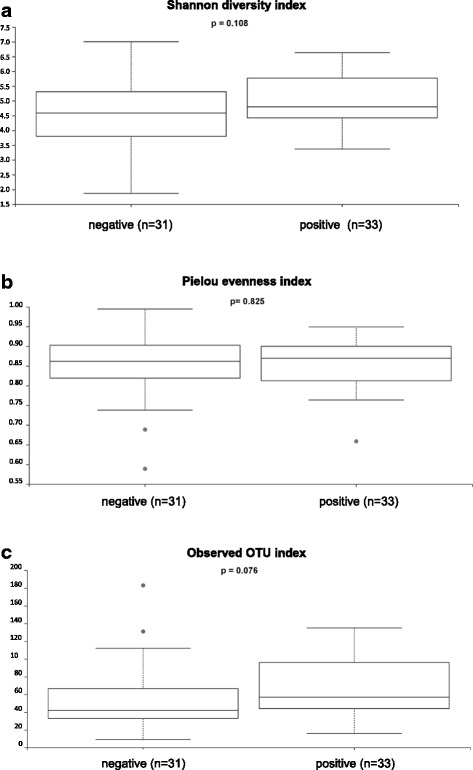
Alpha diversity analysis. Within-sample diversity measured by the Shannon-Index (**a**), Pielou’s measure of species evenness (**b**) and observed OTUs (**c**). Samples were rarefied to a sampling depth of 1000. Kruskal Wallis test was performed to analyze statistical significance

**Fig. 3:**
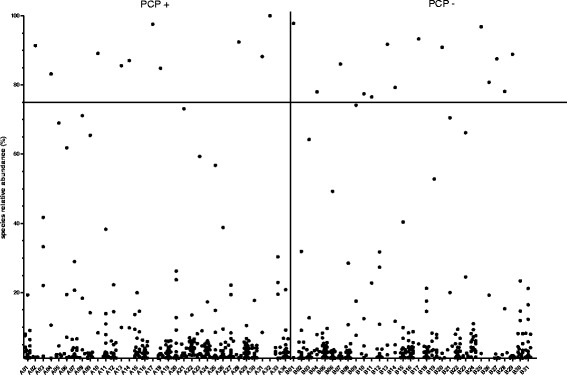
Proportional abundance of individual bacterial species in PCP+ (A01-A34) and PCP- (B01-B31). Each point represents one species (level7). The samples of the individual patients are arrayed along the x-axis. The relative abundance of each species is illustrated on the y-axis. All species that account for at least 1% relative abundance are included. The horizontal bar indicates the 75% threshold

**Fig. 4:**
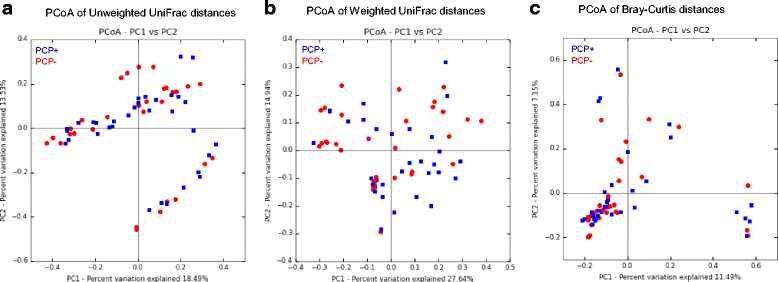
PCoA 2D–plots of unweighted UniFrac distances (**a**), weighted UniFrac distances (**b**) and Bray-Curtis distances (**c**) of bacterial communities of PCP+ (blue) and PCP- (red) patient samples

On class, order family and genus level, no significant differences corrected for multiple testing were observed between PCP- and PCP+ patients. The microbial composition of each sample on phylum and genus level is shown in the Additional file 1: Figure S1 and Additional file 2: Figure S2. The within-sample phylotype richness and evenness (alpha diversity) and dissimilarity between samples (beta diversity) were calculated on a rarefied frequency-feature table with a minimum number of 1000 sequences per sample. No differences in alpha diversity metrics were detected between both patient groups. Shannon diversity index (*p* = 0.108), Pielou’s measure of evenness (pielou_evenness) index (*p* = 0.825), observed_otus index (*p* = 0.076) and Faith’s phylogenetic diversity metric (*p* = 0.506) were not statistically different between PCP+ and PCP- samples (Fig. 2 and Additional file 3: Figure S3). Samples of PCP- and PCP+ patients were not separated or clustered according to PCoA based on weighted and unweighted UniFrac phylogenetic distances, Bray-Curtis distances and Jaccard distances (Fig. 3 and Additional file 4: Figure S4). PCP+ and PCP- samples were not statistically different using permutational multivariate analysis of variance (PERMANOVA) with 999 permutations for all used distance metrics (p = 0.108 for unweighted UniFrac; *p* = 0.182 for weighted UniFrac, *p* = 0.495 for Bray-Curtis; *p* = 0.269 for Jaccard). Furthermore, we analysed if a single species dominates the individual samples by calculating the relative species abundance (Level 7) in each sample. In 10 PCP+ patients and 14 PCP- patients, the microbiome was dominated by a single species with a relative abundance of at least 75% (Fig. 4). The dominating species of the 10 PCP+ samples represented the genera *Enterococcus*, *Streptococcus*, *Staphylococcus*, *Acinetobacter*, *Escherichia, Citrobacter* and *Stenotrophomonas* and the dominating species of the PCP- samples included *Serratia*, *Enterococcus*, *Neisseria, Escherichia*, *Pseudomonas, Stenotrophomonas*, *Legionella*, *Staphylococcus* and *Mycoplasma*, resulting in a total of 12 different genera for these samples (Additional file 5: Table S1).

## Discussion

Pneumocystosis is an opportunistic fungal infection that is associated with a high morbidity and mortality in immunocompromised patients. The natural habitat and the ways of acquisition and transmission of this organism in humans are poorly understood. It is unclear so far if a specific dysbiosis of the lung microbiota may promote uncontrolled colonization and overt disease in immunocompromised patients, like it is suggested for *Aspergillus fumigatus* [8, 17].

Although we found differences in the mean relative abundance of the microbial composition of PCP+ and PCP- patients, these differences were not statistically significant, which was also due to a highly heterogenous microbial composition of the individual PCP+ samples, indicated by high standard deviations for individual taxa, comparable in its extent to the heterogenous group of PCP- patients. Healthy lungs are an ecologically unfavourable environment for most bacteria accompanied by minimal reproduction [18]. The oral microbiome usually is the primary source for the bacterial microbiota of the lungs [19, 20] with *Prevotella*, *Veillonella* and *Streptococcus* as the dominating genera. During critical illness, e.g. during bacterial pneumonia, the environmental conditions in the lungs shift abruptly, resulting in protein-rich fluids in the alveoli, serving as energy source contributing to the changing microbiome of the lungs [12]. A dominant single species usually composes the vast majority of sequences from BAL in bacterial pneumonia [21]. During chronic or acute lung disease other than bacterial pneumonia, a shift towards proteobacteria of the gastrointestinal tract including a loss of diversity has been reported [12]. The composition of the lung microbiome in PCP+ patients with presence of proteobacteria as second most abundant phylum indicates a shift towards the microbiome of critically ill patients with other diseases, although the average abundance of the presence of proteobacteria tends to be lower in PCP+ patients compared with PCP- patients in our study.

All patients included in our study received mechanical ventilation. Mechanical ventilation of critically ill patients alone was shown to be associated with changes in the respiratory microbiome [22, 23], whereas an increased duration of ventilation resulted in a decreased alpha diversity [22, 23]. In addition, the domination of a single taxon was reported in many patients [23]. This observation is in agreement with our data. 29% of the PCP+ and 45% of PCP- patient samples were dominated by a single bacterial species (≥ 75% of the sequence reads). However, and more importantly, we could not find a single bacterial species dominating all PCP+ samples, suggesting that no bacterial co-factor seems to be essential for successful *P. jirovecii* infection.

### Study limitations

The study is limited by its retrospective design. Furthermore, BAL samples were drawn from different departments of the University Hospital Essen and various physicians performing the sampling procedure, so that the procedure itself was not completely standardized. For example, it is unknown if the bronchoscope was passed via the oral or nasal route, which may cause different contamination of the sample with the pharyngeal or nasal bacterial flora. We included BAL samples from PCP+ patients treated during 2013 and 2014, but did not include PCP- patients from these years. Also, in automated DNA extraction procedures, DNA contamination of reagents or eluate may influence microbiome analyses, especially in specimens with low bacterial biomass. Therefore, we excluded the sequence variants which were distributed significantly different between the years 2013–2014 and 2015–2016, as DNA was extracted shortly after sampling. Nevertheless, the removal of these significantly different distributed sequence variants does not represent the proper negative controls and we cannot guarantee that all contaminants have been removed.

In addition, the study lacks of a group-matched analyses that was not possible due to the heterogeneous patients collective both in the PCP+ and PCP- group. The underlying diagnosis, co-morbidities, the immune-status and several other factors of medical treatment can have meaningful effects on the pulmonary microbiome. Furthermore, we did not analyse the effects of antibiotic treatment, in particular the effects of cotrimoxazole administration (standard therapy for pneumocystosis) However, a recent study showed that antibiotic administration in mechanical ventilated patients does not significantly affect the lung microbiome [22].

## Conclusion

The study is the first report analysing the pulmonary microbial communities in intensive care unit patients with pneumocystosis, and comparing them with the lung microbiome of intensive care patients with other diseases. Even though no significant differences in microbial composition between patients with and without pneumocystosis were observed, the current study may be a basis for further works understanding the interaction between *Pneumocystis* and the lung microbial composition.

## Additional files

Additional file 1: Figure S1. Composition of the bacterial community at the phylum level for individual PCP+ and PCP- samples. (DOCX 1968 kb)

Additional file 2: Figure S2. Composition of the bacterial community at the genus level for individual PCP+ and PCP- samples. Legend shows 40 most abundant bacterial genera. (DOCX 2386 kb)

Additional file 3: Figure S3. Alpha diversity analysis. Within-sample diversity measured by Faith’s phylogenetic diversity. Samples were rarefied to a sampling depth of 1000. Kruskal Wallis test was performed to test for statistical significance. (DOCX 165 kb)

Additional file 4: Figure S4. PCoA plots of Jaccard UniFrac distances of bacterial communities of PCP+ (blue) and PCP- (red) patient samples. (DOCX 138 kb)

Additional file 5: Table S1. Table represents samples that are dominated by a single species, with a relative abundance of at least 75%. Genera of these species are specified. (DOCX 30 kb)

## Abbreviations

AIDS: Acquired immunodeficiency syndrome
BAL: Bronchoalveolar lavage
FDR: False discovery rate
ICU: Intensive care unit
PCoA: Principal coordinate analysis
PCP: Pneumocystis jirovecii pneumonia
PERMANOVA: Permutational multivariate analysis of variance
SD: Standard deviation
