# Supplementary figures and images for: The lung microbiome in patients with pneumocystosis
Source: BMC Pulm Med. 2017 Dec 4;17:170. doi: 10.1186/s12890-017-0512-5 (PMC5715545; doi:10.1186/s12890-017-0512-5)

**Additional file 1: Figure S1.**


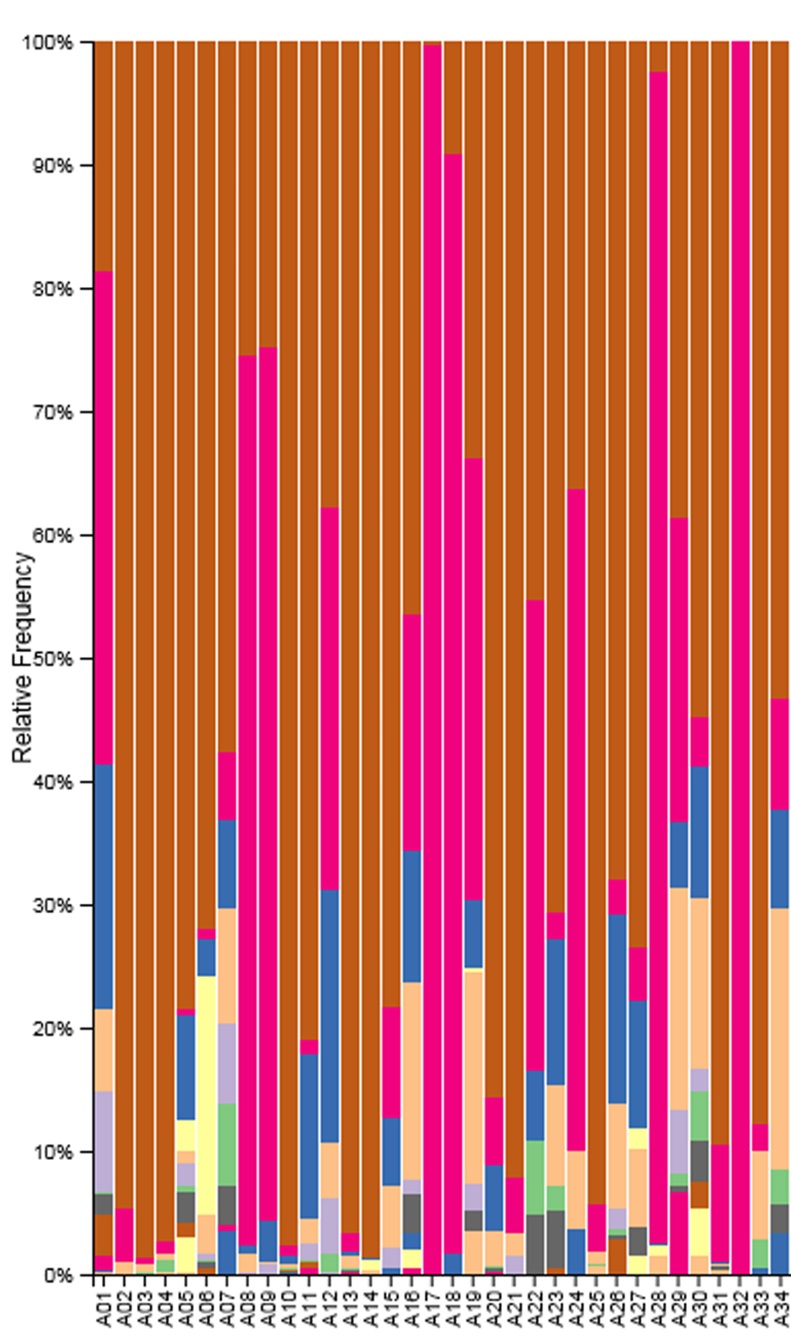

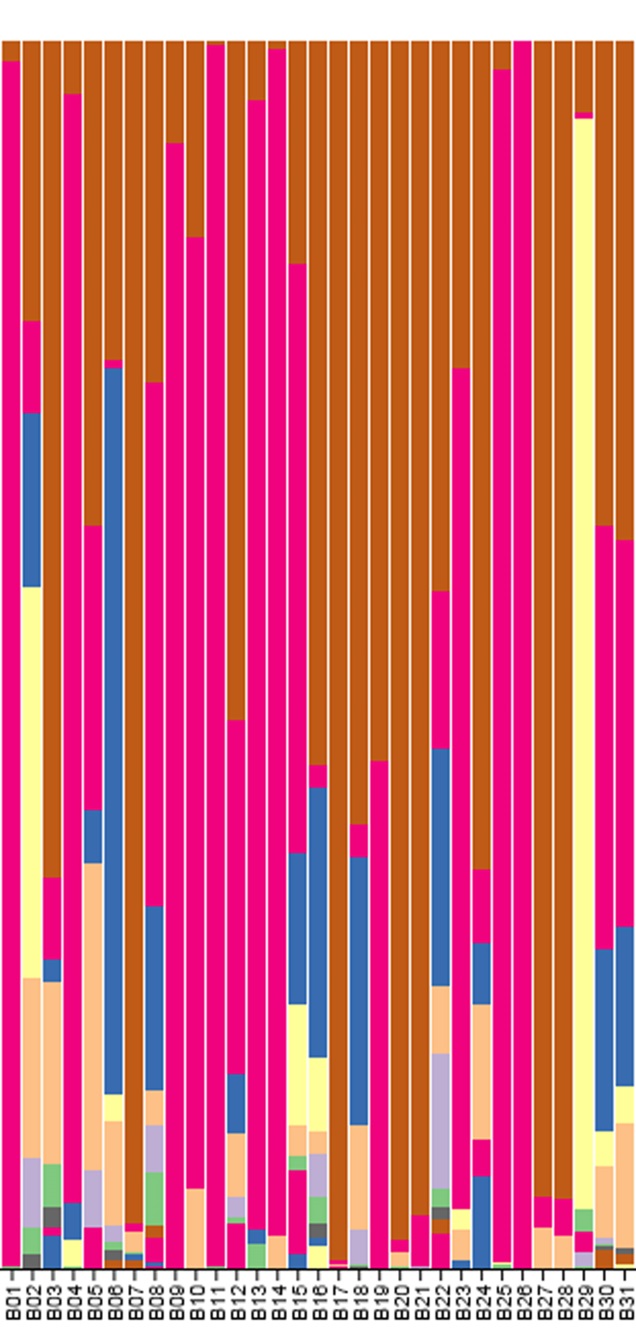


PCP+

PCP-


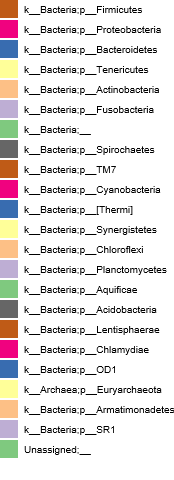

Supplement: Supplementary file 1 — Composition of the bacterial community at the phylum level for individual PCP+ and PCP- samples. (DOCX 1968 kb) [file 12890_2017_512_MOESM1_ESM.docx]

**Additional file 2: Figure S2.**


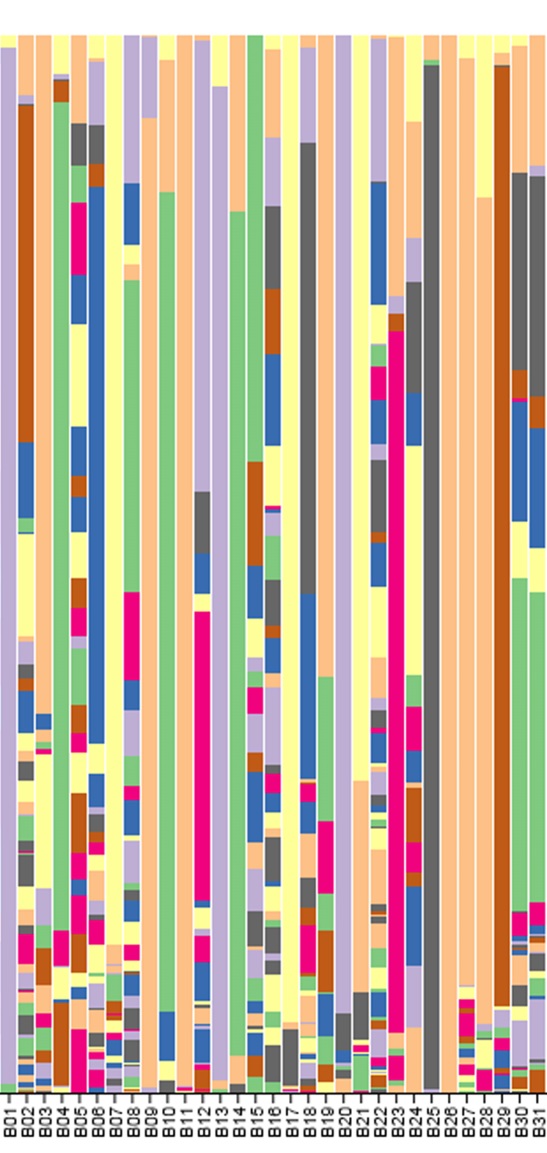

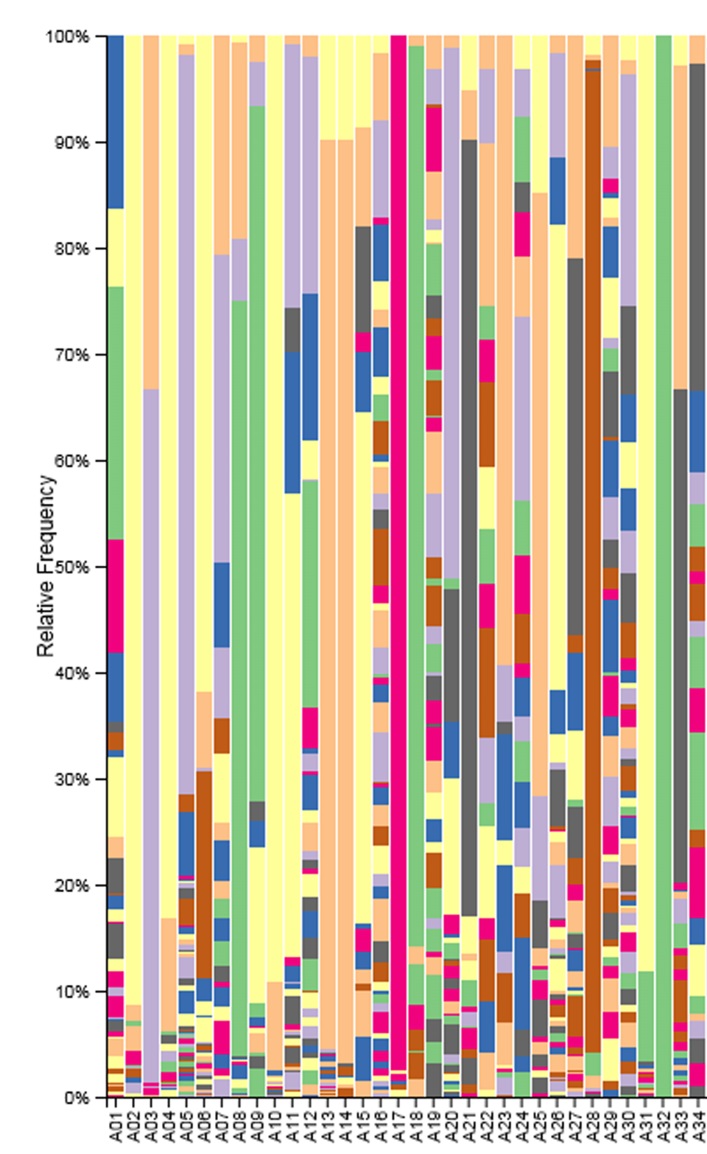


PCP+

PCP-


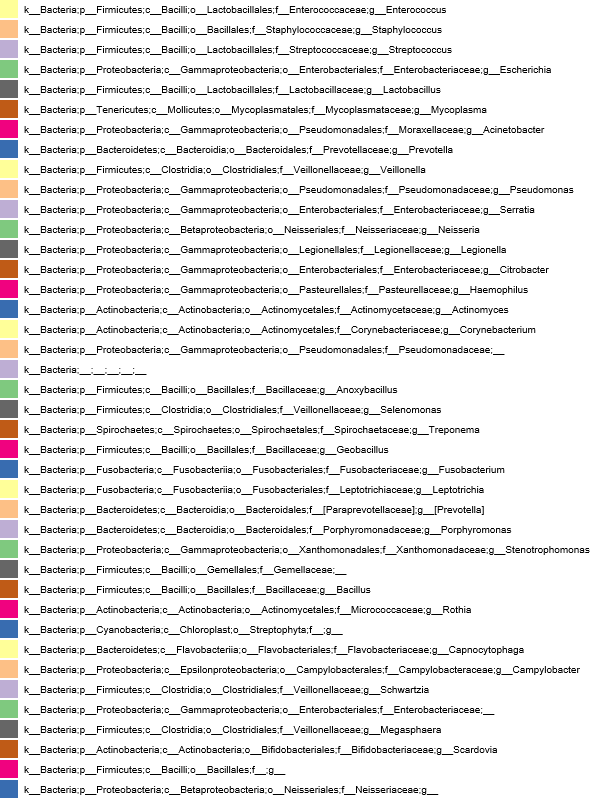

Supplement: Supplementary file 2 — Composition of the bacterial community at the genus level for individual PCP+ and PCP- samples. Legend shows 40 most abundant bacterial genera. (DOCX 2386 kb) [file 12890_2017_512_MOESM2_ESM.docx]

**Additional file 3: Figure S3.**

**Faith´s Phylogenetic diversity**


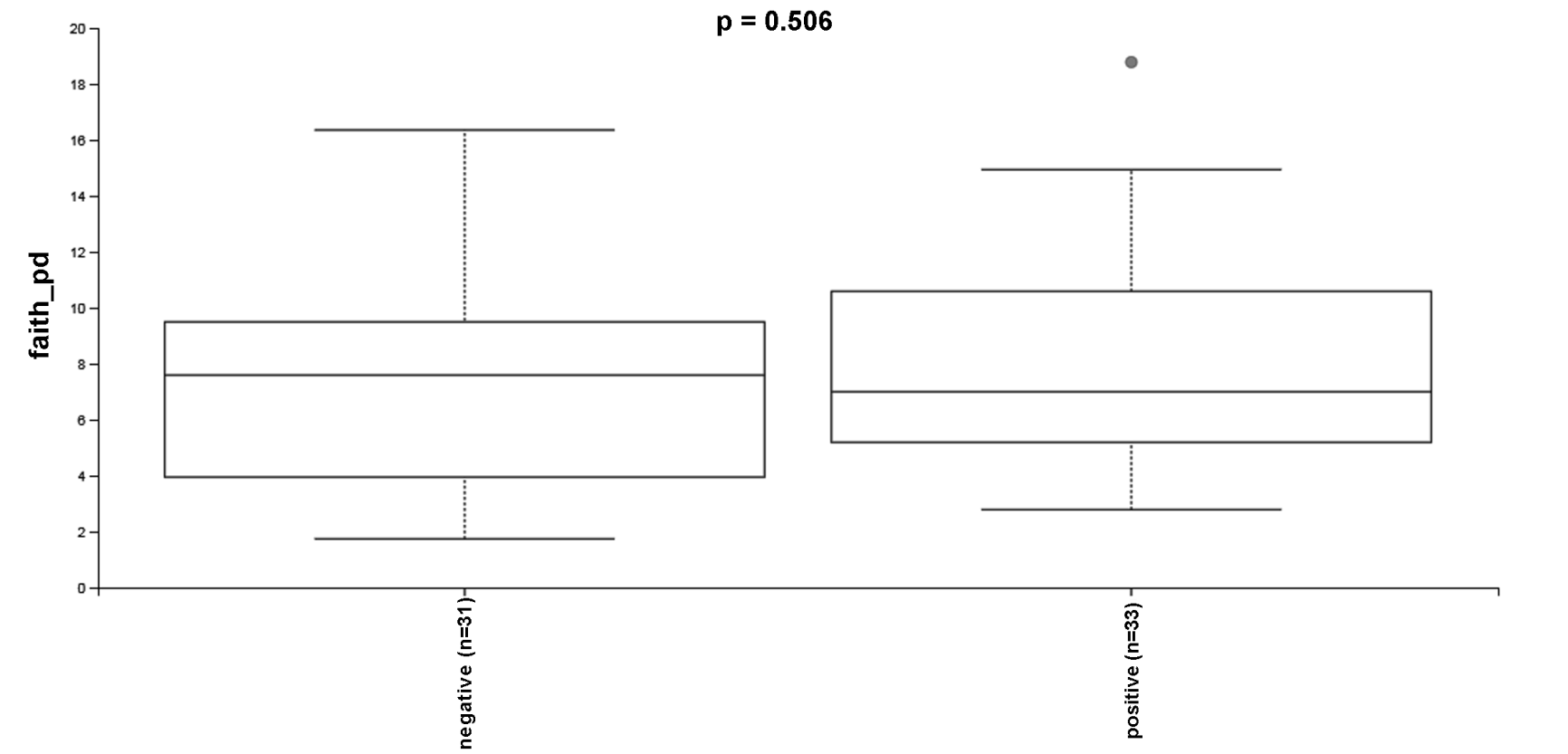


positive (n=33)

negative (n=31)

Supplement: Supplementary file 3 — Alpha diversity analysis. Within-sample diversity measured by Faith’s phylogenetic diversity. Samples were rarefied to a sampling depth of 1000. Kruskal Wallis test was performed to test for statistical significance. (DOCX 165 kb) [file 12890_2017_512_MOESM3_ESM.docx]

**Additional file 4: Figure S4.**

PCoA of Jaccard distances


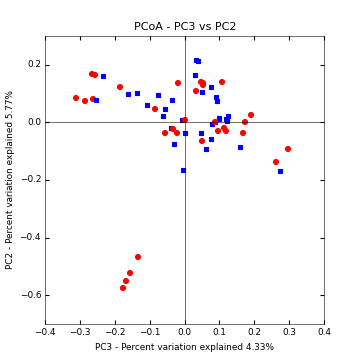

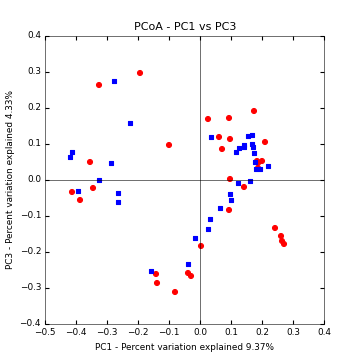


PCP+
PCP-


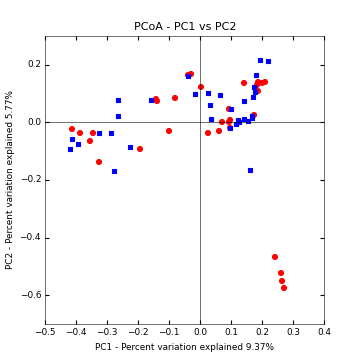


PCP+
PCP-

PCP+
PCP-

Supplement: Supplementary file 4 — PCoA plots of Jaccard UniFrac distances of bacterial communities of PCP+ (blue) and PCP- (red) patient samples. (DOCX 138 kb) [file 12890_2017_512_MOESM4_ESM.docx]
