# Supplementary material for: The lung microbiome in patients with pneumocystosis
Source: BMC Pulm Med. 2017 Dec 4;17:170. doi: 10.1186/s12890-017-0512-5 (PMC5715545; doi:10.1186/s12890-017-0512-5)
Supplement: Supplementary file 5 — Table represents samples that are dominated by a single species, with a relative abundance of at least 75%. Genera of these species are specified. (DOCX 30 kb) [file 12890_2017_512_MOESM5_ESM.docx]

**Additional file 5: Table S1.**

| **Sample** | **relative abundance (%)** | **Genus** |
| --- | --- | --- |
| A02 | 91.4 | *Enterococcus* |
| A04 | 83.2 | *Enterococcus* |
| A10 | 89.1 | *Enterococcus* |
| A13 | 85.6 | *Staphylococcus* |
| A14 | 87.1 | *Staphylococcus* |
| A17 | 97.6 | *Acinetobacter* |
| A18 | 84.8 | *Escherichia* |
| A28 | 92.4 | *Citrobacter* |
| A31 | 88.2 | *Enterococcus* |
| A32 | 100 | *Stenotrophomonas* |
| B01 | 97.8 | *Serratia* |
| B04 | 78.0 | *Neisseria* |
| B07 | 86.1 | *Enterococcus* |
| B10 | 77.4 | *Escherichia* |
| B11 | 76.6 | *Pseudomonas* |
| B13 | 91.8 | *Serratia* |
| B14 | 79.3 | *Stenotrophomonas* |
| B17 | 93.3 | *Enterococcus* |
| B20 | 90.9 | *Streptococcus* |
| B25 | 96.8 | *Legionella* |
| B26 | 80.8 | *Pseudomonas* |
| B27 | 87.5 | *Staphylococcus* |
| B28 | 78.1 | *Staphylococcus* |
| B29 | 88.9 | *Mycoplasma* |
